# Implication of COPB2 Expression on Cutaneous Squamous Cell Carcinoma Pathogenesis

**DOI:** 10.3390/cancers14082038

**Published:** 2022-04-18

**Authors:** Taiqin Chen, Ki-Yeol Kim, Yeongjoo Oh, Hei Cheul Jeung, Kee Yang Chung, Mi Ryung Roh, Xianglan Zhang

**Affiliations:** 1 Department of Dermatology, Yanbian University Hospital, Yanji 133000, China; taegeum2013@yuhs.ac; 2 Department of Dental Education, BK21 PLuS Project, Yonsei University College of Dentistry, Seoul 03722, Korea; kky1004@yuhs.ac; 3 Department of Dermatology, Yongin Severance Hospital, Yonsei University College of Medicine, Seoul 16995, Korea; ohyjune@yuhs.ac; 4 Cancer Metastasis Research Center, Division of Medical Oncology, Cancer Center Gangnam Severance Hospital, Yonsei University College of Medicine, Seoul 06273, Korea; jeunghc1123@yuhs.ac; 5 Department of Dermatology, Cutaneous Biology Research Institute, Severance Hospital, Yonsei University College of Medicine, Seoul 03722, Korea; kychung@yuhs.ac; 6 Department of Dermatology, Gangnam Severance Hospital, Cutaneous Biology Research Institute, Yonsei University College of Medicine, Seoul 06273, Korea; 7 Department of Pathology, Yanbian University Hospital, Yanji 133000, China; 8 Oral Cancer Research Institute, Yonsei University College of Dentistry, Seoul 03722, Korea

**Keywords:** COPB2, cSCC, pathogenesis, tumor immune microenvironment

## Abstract

**Simple Summary:**

The present study aimed to evaluate the effect of COPB2 expression on cutaneous squamous cell carcinoma (cSCC) pathogenesis. cSCC, a common category of skin cancer, is marked by a reasonably favorable prognosis. However, there has been a steady rise in the annual incidence of cases; in particular, a subset of cases showed aggressive progression. However, the underlying molecular mechanism of cSCC pathogenesis is largely unknown. In the present study, we found that COPB2 may act as a potential oncogene and modulator of the tumor immune microenvironment in cSCC pathogenesis. Therefore, COPB2 can serve as a novel predictive prognostic biomarker and immunotherapeutic target in cSCC patients.

**Abstract:**

The underlying molecular mechanisms of cutaneous squamous cell carcinoma (cSCC) pathogenesis are largely unknown. In the present study, we aimed to evaluate the effect of coatomer protein complex subunit beta 2 (COPB2) expression on cSCC pathogenesis. Clinicopathological significance of COPB2 in cSCC was investigated by analyzing the Gene Expression Omnibus (GEO) database and through a retrospective cohort study of 95 cSCC patients. The effect of COPB2 expression on the biological behavior of cSCC cells was investigated both in vitro and in vivo. We found that COPB2 expression was significantly higher in cSCC samples than in normal skin samples. In our cohort, a considerable association was found between COPB2 expression and indicators of tumor immune microenvironment (TIME), such as histocompatibility complex class (MHC) I, and MHC II, CD4+/ CD8+ tumor-infiltrating lymphocytes. Additionally, COPB2 expression had an independent impact on worsened recurrence-free survival in our cohort. Furthermore, decreased proliferation, invasion, tumorigenic activities, and increased apoptosis were observed after *COPB2* knockdown in cSCC cells. COPB2 may act as a potential oncogene and candidate modulator of the TIME in cSCC. Therefore, it can serve as a novel predictive prognostic biomarker and candidate immunotherapeutic target in cSCC patients.

## 1. Introduction

Coat proteins (COPs) contribute to the movement of proteins and lipids between the Golgi and endoplasmic reticulum (ER) through the formation of transport vesicles during both physiological and pathological processes in numerous types of eukaryotic cells, including cancer cells. COPs can be divided into three types: clathrin, COPI, and COPII [1]. The coatomer protein complex subunit beta 2 (COPB2) is one of the seven subunits that form COPI.

Recently, COPB2 has been shown to execute a range of oncogenic activities in various types of cancers. In lung adenocarcinoma cells, COPB2 overexpression attenuated apoptosis and promoted both proliferation and tumorigenesis via upregulation and nuclear translocation of YAP1 in vitro [2]. Moreover, COPB2 can induce G0/G1 and G2/S phase arrest in various types of cancers [1,3,4]; the regulatory action of COPB2 on the contributors of cell-cycle progressions, such as cyclin A, p21, and p16, was indicated as the underlying molecular mechanism of cell-cycle arrest in human colon cancer [1]. Furthermore, the receptor tyrosine kinase (RTK), c-Jun N-terminal kinase (JNK)/c-Jun signaling pathways, as well as certain microRNAs, such as miR-4461, miR-335-3p, and miR-216a-3p, have been delineated as molecular mechanisms related to the oncogenic functions of COPB2 in cancer progression [5,6,7,8,9]. In addition, a retrospective cohort study found that COPB2 overexpression is significantly correlated with poor prognosis in patients with various types of cancers, including lung adenocarcinoma, hepatocellular carcinoma, and glioma; consequently, it was proposed as a possible prognostic and theranostic marker for cancer patients [2,10,11].

The crucial role of the tumor immune microenvironment (TIME) in cancer progression has been widely established, and the prognostic impact of the density of infiltrating tumor immune cells, such as CD4^+^/CD8^+^ T cells, natural killer (NK) cells, regulatory T cells (Tregs), myeloid-derived suppressor cells (MDSCs), and tumor-associated macrophages (TAMs), is a central concept in TIME study [12,13,14]. In the late 1980s, some investigators showed that increased lymphocytic infiltration was associated with favorable outcomes in patients with rectal carcinoma and melanoma [15,16]. The tumor-promoting or tumor-suppressive impact of various types of infiltrating immune cells of tumor tissues has been demonstrated in subsequent retrospective cohort studies in numerous types of cancers. Therefore, the underlying molecular mechanisms mediating immune cell recruitment in cancer tissues have received considerable attention in contemporary cancer research. Recently, according to the bioinformatical analysis of the public database, such as GSE16011, some investigators found that the expression status of the COPB2 transcript was significantly related to the recruitment of immune cells, the major histocompatibility complex (MHC); they postulated that the regulatory action of COPB2 on TIME may be a crucial molecular contributor to its clinicopathological significance in cancer progression [10].

As a common skin cancer, cutaneous squamous cell carcinoma (cSCC) has a generally favorable treatment outcome. However, its incidence is increasing annually, with a subset of cases showing postoperative recurrence, and the recurrence of cSCC often shows more aggressive progression than the primary tumors [17,18,19,20]. The well-known etiological risk factors for cSCC include ultraviolet or arsenic exposure, human papillomavirus infection, and immunosuppression [21,22]. However, the underlying molecular mechanism of cSCC remains unclear. In the present study, we aimed to evaluate the impact of COPB2 expression on the pathogenesis of cSCC. We found that COPB2 exerts adverse prognostic effects and possible oncogenic activity in cSCC. Moreover, a considerable association was found between COPB2 expression and the indicators of TIME, such as MHC class I (MHC I), MHC class II (MHC II), CD4^+^ tumor-infiltrating lymphocytes (TILs), and CD8^+^ TILs in our cohort. COPB2 may play crucial roles in cSCC pathogenesis and may serve as a novel candidate biomarker of cSCC.

## 2. Materials and Methods

### 2.1. Clinical Samples

A total of 95 patients with cSCC were selected for the present cross-sectional retrospective cohort study. All patients were diagnosed with cSCC at the Department of Pathology, Yonsei University Health System, Seoul, South Korea, from 2000 to 2010. Five normal skin tissue samples adjacent to cSCC were also included in the present study. The clinicopathological characteristics of the patients are presented in Table 1. The study design was approved by the institutional review board of the Yonsei University Health System, Severance Hospital (IRB 2018-0874-001).

### 2.2. Establishment of COPB2 Knockdown cSCC Cells In Vitro

Two human cSCC cell lines, A431 and HSC-1, were purchased from the Korean Cell Line Bank (Seoul, South Korea) and the Japanese Collection of Research Bioresources Cell Bank (Osaka, Japan), respectively, for in vitro and in vivo analyses. *COPB2* knockdown stable HSC-1 and A431, as well as related control cells, were established using pGFP-C-shCOPB2 lentiviral particles (OriGene, Rockville, MD, USA) and maintained in Roswell Park Memorial Institute (RPMI) 1640 medium supplemented with 10% fetal bovine serum (FBS; Invitrogen, Waltham, MA, USA) and 1% streptomycin/penicillin (Gibco, Waltham, MA, USA).

### 2.3. Trypan Blue Assay

To determine the proliferation ability of each group of cells, a trypan blue assay was performed. Each group of cells was seeded in 6-well plates (1 × 10^4^/well) and the number of cells was counted at each indicated time point after trypan blue staining.

### 2.4. Matrigel Invasion Assay

Matrigel invasion assay was used herein to determine the invasion ability of each group of cells. Transwell inserts (BD Biosciences, Bedford, MA, USA) with 8 μm pore size were used after Matrigel (BD Biosciences, San Jose, CA, USA) coating. The culture medium for the upper and lower chambers was supplemented with 2% bovine serum albumin (Sigma-Aldrich, St. Louis, MO, USA) and 20% FBS. Each group of cells was seeded in the upper chamber at 4 × 10^5^ density and invading cells were counted after 34 h of culture under visualization with 0.25% crystal violet (Sigma-Aldrich, St. Louis, MO, USA).

### 2.5. Cell Apoptosis Assay

The number of apoptotic cells in each group of cSCC cells was determined using annexin V-FITC and propidium iodide (PI) staining kit (BD Biosciences), according to the manufacturer’s protocol. The staining patterns were analyzed using flow cytometry (BD Biosciences) and Cell Quest Software (BD Biosciences). Apoptotic cells in the tumor mass obtained from the xenograft mouse model were highlighted using a TACS2 TdT-DAB In Situ Apoptosis Detection Kit (R&D systems, Minneapolis, MN, USA), according to the manufacturer’s protocol, and counted under a microscope.

### 2.6. In Vivo Xenograft Mouse Model

Five-week-old female BALB/c-*nu/nu* mice (NARA Biotech, Seoul, Korea) (n = 10) were randomly divided into two groups for in vivo analysis. A total of 5 × 10^6^ cells from each group of cells was suspended in 100 μL phosphate-buffered saline and subcutaneously injected into the right flank of the two groups of mice. The volume of tumor nodules was calculated after 7 days of injection, every 3 days, based on the width and length according to a previous study [23]. After 22 days, the mice were sacrificed, and the tumor nodules were removed for further analysis.

### 2.7. Immunochemical Staining

The paraffin blocks of the tissue samples and each group of cells were cut into 4-micrometer sections for immunohistochemistry. After deparaffinization and rehydration, antigen retrieval and blocking of endogenous peroxidase activity were performed for the sections, as described in a previous study [24]. Each group of cells was seeded in the chamber slide (Thermo Scientific, Waltham, MA, USA) for immunocytochemistry, cultured for 24 h, and subsequently fixed with 95% ethanol for 30 min at room temperature (RT). COPB2 (rabbit polyclonal IgG, working dilution 1/50, Abnova, PAB23074, Walnut, CA, USA), MHC I (rabbit monoclonal IgG, working dilution 1/1000, Abcam, Cambridge, UK, ab134189), MHC II (rabbit monoclonal IgG, working dilution 1/1000, Abcam, ab55152), CD4 (rabbit monoclonal IgG, working dilution 1/1000, Abcam, ab133616), and CD8 (rabbit monoclonal IgG, working dilution 1/1000, Abcam, ab108343) antibodies were used as primary antibodies herein. After incubation with the secondary antibody, Real Envision HRP Rabbit/Mouse Detection System (Dako, Santa Clara, CA, USA), for 30 min at RT, the sections were visualized with 3,3′-diaminobenzidine.

The total immunoreactivity of COPB2, MHC I, and MHC II for the sections was calculated based on the staining intensity and the percentage of the cells, which were further divided into two groups: low-expression and high-expression groups, as described in a previous study [24]. The TILs were counted in two regions: tumors (intratumoral TILs) and stromal TILs (peri-tumoral TILs). Three hot spots of TILs were picked in each region of the tissue sections at 200× magnification. The number of TILs was calculated as the mean value of the total TIL count of the three hot spots. The patients were subdivided into two groups according to the mean TIL count: low-TIL group and high-TIL groups.

### 2.8. Statistical Analysis

All statistical analyses were performed using R package version 3.5.1 (The R Foundation for Statistical Computing, Vienna, Austria). The correlation between MHC and COPB2 transcript expression was detected using canonical correlation analysis. The difference in COPB2 transcript expression between normal and tumor tissues was analyzed using Fisher’s exact test. The influence of *COPB2* knockdown on the biological behavior of cSCC cells was analyzed using the Mann-Whitney U-test. The chi-square test, Fisher’s exact test, and multivariate cox regression analysis were used to investigate the clinicopathological significance of COPB2 expression in the cSCC cohort.

## 3. Results

### 3.1. COPB2 Knockdown Largely Influenced the Biological Behavior of cSCC Cells In Vitro

**Figure 1:**
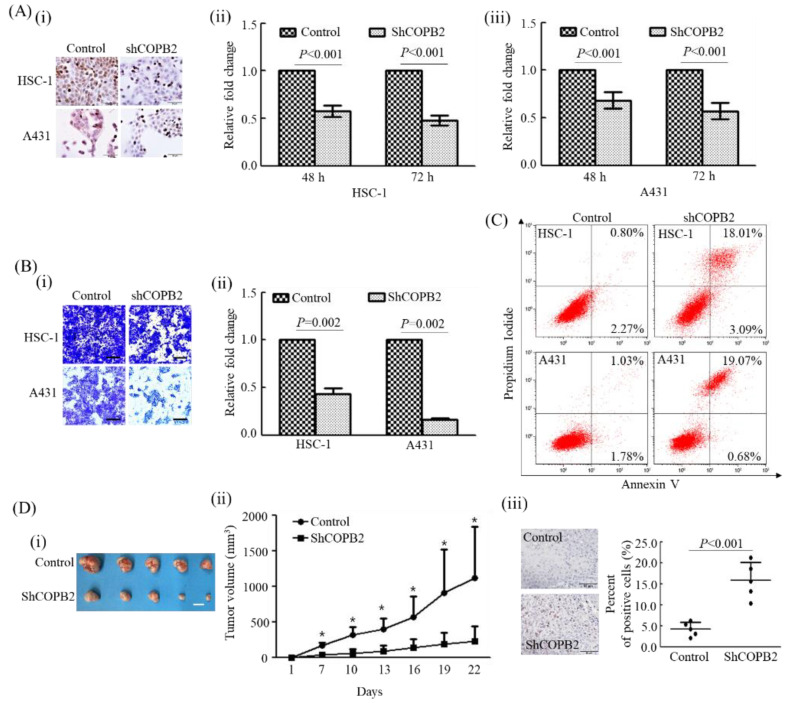
Effect of *COPB2* knockdown on biologic behavior of cutaneous squamous cell carcinoma (cSCC) cells: (**A**) (i) COPB2 protein expression decreased predominantly after *COPB2* knockdown in both HSC-1 and A431 cells (Magnification, 400×; Scale bar, 50 μm); (ii) Proliferation ability of both HSC-1 and A431 cells decreased significantly after *COPB2* knockdown at the indicated time points (Both *p* < 0.001, Mann-Whitney U-test). (**B**) (i) Representative patterns of cell invasion in each group of cSCC cells (Magnification, 100×; Scale bar, 200 μm). (ii) Invasion ability of both HSC-1 and A431 cells decreased significantly after *COPB2* knockdown (Both *p* = 0.002, Mann-Whitney U-test). (**C**) Apoptotic cells increased predominantly after *COPB2* knockdown in both HSC-1 and A431 cells. (**D**) (i) Tumor nodules of xenograft mouse models obtained from control and shCOPB2 groups of A431 cells (Scale bar, 1 cm). (ii) Volume of the tumor nodules increased significantly in control group compared to that in the *COPB2* knockdown group at the indicated days (* *p* = 0.008, Mann-WhitneyU-test). (iii) Representative patterns of apoptotic cells in tumor nodules from each group of A431 cells-xenograft models (Magnification, 400×; Scale bar, 50 μm). Apoptotic cells were predominantly higher in the *COPB2* knockdown group than in the control group in A431 cells (*p* < 0.001, Mann-Whitney U-test).

To investigate the influence of *COPB2* knockdown on the proliferation of cSCC cells, the number of HSC-1 and A431 cells was comparatively investigated in the control and COPB2-shRNA-transfected groups at each indicated time point. The number of cells was significantly higher in the control groups than in the COPB2-shRNA-transfected group at the time points of 48 h (1.75- and 1.46-fold, respectively) and 72 h (2.08- and 1.75-fold, respectively, in both HSC-1 and A431 cells (Figure 1A i–iii).

The influence of *COPB2* knockdown on the invasion ability of cSCC cells was investigated using a Matrigel invasion assay. The cells that traversed the membrane were 2.29- and 6.16-fold higher in the control group than in COPB2-shRNA-transfected group both in HSC-1 and A431 cells (Figure 1B i,ii). Moreover, apoptotic cells were predominantly increased in the COPB2-shRNA-transfected group (21.10% and 19.75%) than in the control group (3.07% and 2.81%) in both HSC-1 and A431 cells (Figure 1C).

### 3.2. COPB2 Knockdown Attenuated Tumorigenic Activity In Vivo

To investigate the influence of *COPB2* knockdown on the tumorigenic activity of cSCC cells, the volume of tumor nodules from xenograft mouse models was compared between the control and *COPB2* knockdown groups in A431 cells (Figure 1D i,ii). The volume of tumor nodules was significantly decreased in the *COPB2* knockdown group compared with that in the control group in A431 cells. The number of apoptotic cells was predominantly higher in the *COPB2* knockdown group than in the control group in A431 cells (*p* = 0.008) (Figure 1D iii).

### 3.3. Clinicopathologic Significance of COPB2 Expression in cSCC

**Figure 2:**
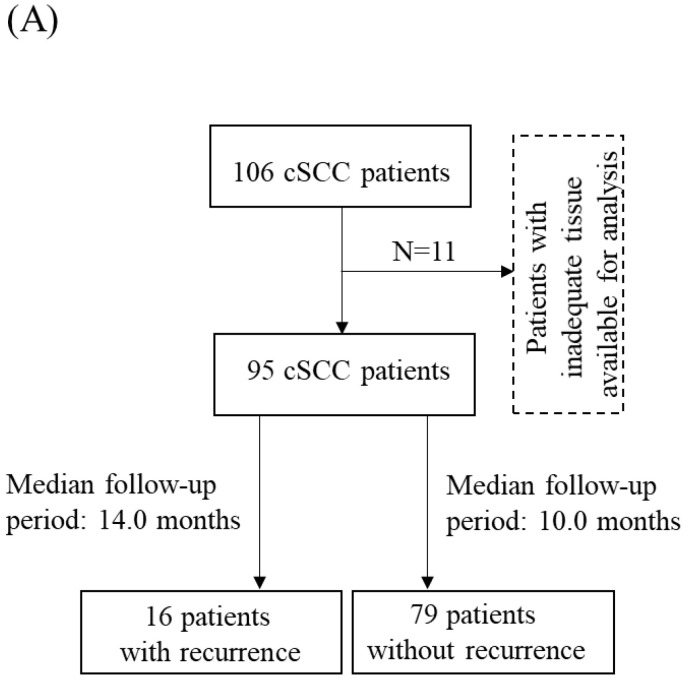

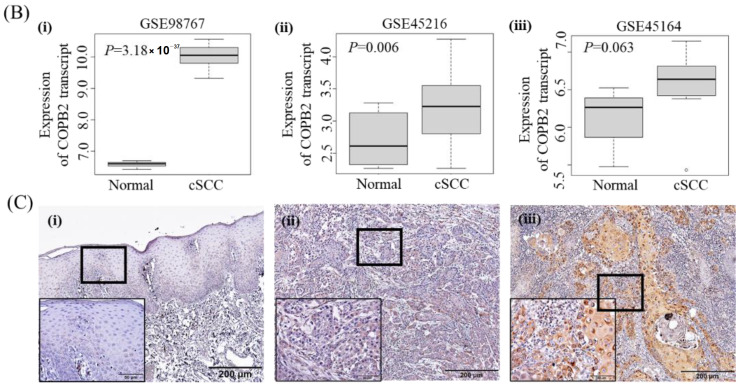
COPB2 expression in normal skin and cSCC samples: (**A**) Flow diagram for selection and outcome of cSCC patients. (**B**) Expression of COPB2 transcript was higher in cSCC than in normal skin samples in the public datasets (i) GSE98767, (ii) GSE45216, and (iii) GSE45164). (**C**) Representative expression patterns of COPB2 expression in (i) normal skin, and (ii) low and (iii) high immunoreactive cSCC tissue samples (Original magnification, 100×; scale bar, 200 μm; magnification for inset, 400×; scale bar for inset, 50 μm).

To investigate the clinicopathologic significance of COPB2 protein expression in cSCC pathogenesis, 11 patients with inadequate tissue available for analysis were excluded from this study, 95 cSCC patients of a total of 106 cSCC patients were selected for further analysis after excluding 11 patients due to inadequate tissue for analysis. Among the 95 patients, 16 patients had cSCC recurrence (median follow-up period: 14.0 months), while 79 patients did have cSCC recurrence (median follow-up period: 10.0 months) during the follow-up period (Figure 2A). According to a bioinformatic analysis of the public datasets (GSE98767, GSE45216, and GSE45164), we found that the expression level of the COPB2 transcript was higher in cSCC than in normal skin samples (*p* < 0.001, *p* = 0.006, and *p* = 0.063, respectively) (Figure 2B). We further compared COPB2 protein expression between five normal skin samples and ninety-five cSCC tissue samples. All the normal skin tissue samples showed low immunoreactivity against COPB2 and the positive cells were restricted in the lower third of the epithelium. In contrast, 22 (23.2%) cSCC patients showed high COPB2 expression, while 73 (76.8%) cSCC patients showed low COPB2 expression. Representative expression patterns of COPB2 in normal skin and cSCC tissue samples are shown in Figure 2C.

**Figure 3:**
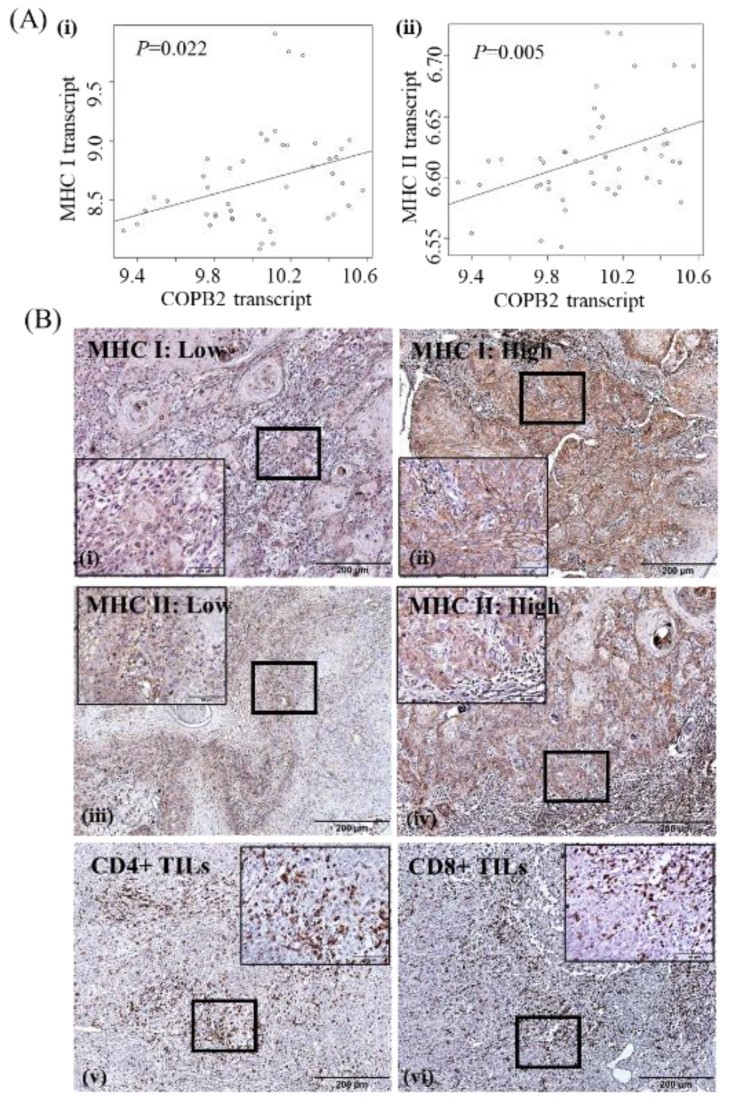
Expression of tumor immune microenvironment (TIME) indicators in cSCC: (**A**) COPB2 expression showed a positive correlation with the expression of both (i) MHC I and (ii) MHC II transcripts in cSCC cell lines in the GSE98767 dataset; (**B**) Representative expression patterns of MHC I, MHC II, and hot spots of CD4+/CD8+ tumor-infiltrating lymphocytes (TILs) in cSCC tissues (Original magnification, 100×; scale bar, 200 μm; magnification for inset, 400×; scale bar for inset, 50 μm).

According to bioinformatic analysis of the public dataset (GSE98767), we found that COPB2 expression showed a positive correlation with the expression levels of both MHC I and MHC II transcripts in cSCC cell lines (Figure 3A). We further evaluated MHC I and MHC II protein expression in 95 cSCC tissue samples. MHC I and MHC II protein expression were found in various types of cells, and only tumor MHC protein expression was investigated herein. Membrane and cytoplasmic expression of MHC I and MHC II was found in 29 (30.5%) and 50 (52.6%) cSCC tissue samples, respectively. Immune reactivity was high in 17 (17.9%) and 27 (28.4%) cSCC tissue samples and low in 78 (82.1%) and 68 (71.6%) cSCC tissue samples against MHC I and MHC II, respectively. In our cohort, MHC I and MHC II expression showed an increasing trend in cSCC tissues with high COPB2 expression (31.8% and 45.5%, respectively) than in those with low COPB2 expression (13.7% and 23.3%, respectively) (*p* = 0.063 and *P* = 0.059, respectively) (Table 2). In addition, intra-and peri-tumoral CD4+ TILs and intra- and peri-tumoral CD8+ TILs were significantly increased in cSCC tissues with high COPB2 expression (59.1%, 68.2%, 63.6%, and 72.7%, respectively) than in cSCC tissues with low COPB2 expression (19.2%, 39.7%, 27.4%, and 37.0%, respectively) (*p* = 0.001, *p* = 0.028, *p* = 0.004, and *p* = 0.006, respectively). No significant association was found between COPB2 expression and clinicopathological characteristics, such as age, gender, lesion site, tumor size, and histologic grade herein (Table 2). Representative expression patterns of MHC I and MHC II, and hot spots of CD4+/CD8+ TILs are shown in Figure 3B.

Representative expression patterns of MHC I and MHC II as well as hot spots of CD8+TILs and CD4+TILs are shown in Figure 3B.

In a univariate cox regression analysis, increased intratumoral CD4+/CD8+TILs, high MHC I, high MHC II, and high COPB2 expression were related to worse recurrence-free survival in our cohort (*p* = 0.013, *p* = 0.048, *p* = 0.015, *p* = 0.012, and *p* < 0.001, respectively) (Table 3). In a multivariate cox regression analysis using age, sex, lesion site, tumor size, histological grade, CD4+ TILs, CD8+ TILs, MHC I, MHC II, and COPB2 as co-factors, larger tumor size (odds ratio [OR]= 4.812, 95% confidence interval [CI]= 1.025–22.588; *p* = 0.046, poorly differentiated histologic grade (OR = 13.576; 95% CI = 1.151–160.104; *p* = 0.038), and high COPB2 expression (OR = 10.905; 95% CI = 1.714–69.372; *p* = 0.011) had an independent impact on the worsened recurrence-free survival of cSCC patients (Table 3).

## 4. Discussion

Increased expression and vital oncogenic roles of COPB2 in cancer progression have been elucidated in various types of cancers, such as breast cancer, gastric cancer, prostate cancer, and glioma [3,5,10,25]. However, the expression status and impact of COPB2 on cSCC progression have not been evaluated. In the present cross-sectional and retrospective cohort study, we evaluated the clinicopathological significance of COPB2 expression in the cSCC cohort. Consistent with other cancers, we found that COPB2 expression was significantly increased in cSCC tissues compared with that in normal skin tissue samples in both the public database and our cohort. In lung adenocarcinoma, patients with increased COPB2 expression showed advanced clinical symptoms more often, such as low pathological grade, presence of lymph node metastasis, higher TNM stage, presence of distant metastasis, and worse overall survival [2]. Moreover, increased COPB2 expression is linked to higher pathological grading in colon cancer and lymph node metastasis in breast cancer [1,25]. Consistent with these findings, increased COPB2 expression was significantly associated with worse recurrence-free survival in patients with cSCC in our cohort. Correspondingly, decreased proliferation and invasion abilities and increased apoptotic activity were found in cSCC cells after *COPB2* knockdown in vitro. Moreover, tumorigenic ability was also hindered after *COPB2* knockdown in cSCC cells. These results implied that, similar to other cancers, COPB2 executes possible oncogenic activities in cSCC progression.

Genetic alterations in cancer cells can trigger immune responses during cancer progression, and tumor-specific antigens are represented on the cell membrane by the MHC. According to a previous study, there is a significant association between COPB2 expression and both MHC I and MHC II in gliomas [26]. In the present study, we also found a significant association between the expression of COPB2 and both MHC I and MHC II transcripts in cSCC cell lines, according to public database analysis. Moreover, in cSCC tissue samples, MHC I and MHC II expression demonstrated an increasing trend in patients with high COPB2 expression than in patients with low COPB2 expression.

MHC I and MHC II can be recognized by cytotoxic CD8+ T cells and CD4+ helper T cells, respectively, and consequently trigger immune responses such as anticancer immunity during cancer progression [26]. In the present study, increased CD8+ or CD4+ TILs were frequently found in both the intra- and peri-tumoral regions of cSCC tissue samples. Similar to a study on glioma, a significant association was found between COPB2 expression and both CD8+ and CD4+TILs in cSCC tissues [10]. In glioma, COPB2 expression was also correlated with the accumulation of Tregs, neutrophils, and MDSCs, which are considered mediators of the immunosuppressive microenvironment in various types of cancers. In addition, certain immune reaction-related pathways, such as antigen processing and presentation, B-cell receptor signaling pathway, and T-cell receptor signaling pathway, were also found to be significant pathways responsible for COPB2 increase in glioma [10]. Therefore, COPB2 may act as a potential modulator of the TIME in various types of cancers, including cSCC.

A significant association between the TIME and cancer progression has been reported in many types of cancers [27,28,29]. In the present study, we found that intratumoral CD4+/CD8+ TILs were significantly increased in patients with worsened outcomes. Generally, cytotoxic CD8+ T cells and CD4+ helper T cells exhibit antitumor effects and are associated with favorable prognoses in various types of cancers. However, immune cells can develop an altered subset via their plasticity in response to the dynamics of the tumor microenvironment during cancer progression, and consequently exhibit aberrant functions in various types of cancers [30]. Unlike classic CD8+ TILs, increased density of CD8^+^ TILs was indicated as a poor prognostic factor in non–small cell lung cancer, renal cell carcinoma, and hepatocellular carcinoma patients [31,32,33]. In addition to the prognostic impact of the TIME, the importance of the TIME in effective cancer immunotherapy has also been highlighted [34]. Hence, modulation of the TIME is a key challenge in cancer therapy. Therefore, further studies are needed to investigate the detailed functions and related molecular mechanisms of COPB2, a possible modulator of the TIME, in cSCC pathogenesis.

## 5. Conclusions

COPB2 may act as a potential oncogene and candidate modulator of the TIME in cSCC pathogenesis. Therefore, it can serve as a novel predictive prognostic biomarker and candidate immunotherapeutic target in cSCC patients.

## Figures and Tables

**Table 1:** Demographics of 5 normal and 95 cSCC patients.

Clinicopathological Variables	No. Patients (%)
Total cases of normal skin tissue	5
Median age, years	43 (32–89)
Gender	
Male	2 (40)
Female	3 (60)
Location	
Head and neck	1 (20)
Acral	1 (20)
Extremity	2 (40)
Trunk	1 (20)
Total cases of cSCC patients	95
Age, years	
Median age (range)	76 (30–98)
≤60	12 (12.6)
>60	83 (87.4)
Gender	
Male	41 (43.2)
Female	54 (56.8)
Location	
Head and neck	79 (83.2)
Acral	12 (12.6)
Extremity	2 (2.1)
Trunk	2 (2.1)
Tumor size, cm	
≤2cm	62 (65.3)
>2cm	33 (34.7)
Histologic grade	
WD	54 (56.8)
MD	32 (33.7)
PD	9 (9.5)
Recurrence	
Yes	16 (16.8)
No	79 (83.2)

WD: Well differentiated; MD: Moderately differentiated; PD: Poorly differentiated.

**Table 2:** Clinicopathologic significance of COPB2 expression in 95 cSCC patients.

Variables	Total, *n* (%)	COPB2		*p* Value
		Low	High	
Age				
≤60	12 (12.6)	7 (58.3)	5 (41.7)	
>60	83 (87.4)	66 (79.5)	17 (20.5)	0.141
Gender				
Male	41 (43.2)	29 (70.7)	12 (29.3)	
Female	54 (56.8)	44 (81.5)	10 (18.5)	0.232
Lesion site				
Head and neck	79 (83.2)	60 (75.9)	19 (24.1)	1.000
Acral	12 (12.6)	9 (75.0)	3 (25.0)	
Extremity	2 (2.1)	2 (100)	0(0)	
Trunk	2 (2.1)	2 (100)	0(0)	
Tumor size, cm				
≤2	62 (65.3)	46 (74.2)	16 (25.8)	0.454
>2	33 (34.7)	27 (81.8)	6 (18.2)	
Histologic grade				
WD	54 (56.8)	40 (74.1)	14 (25.9)	0.872
MD	32 (33.7)	26 (81.3)	6 (18.8)	
PD	9 (9.5)	7 (77.8)	2 (22.2)	
MHC I				
Low	78 (82.1)	63 (80.8)	15 (19.2)	0.063
High	17 (17.9)	10 (58.8)	7 (41.2)	
MHC II				
Low	68 (71.6)	56 (82.4)	12 (17.6)	0.059
High	27 (28.4)	17 (63.0)	10 (37.0)	
Intra CD4+TIL				
Low	68 (71.6)	59 (86.8)	9 (13.2)	0.001
High	27 (28.4)	14 (51.9)	13 (48.1)	
Peri CD4+TIL				
Low	51 (53.7)	44 (86.3)	7 (13.7)	0.018
High	44 (46.3)	29 (65.9)	15 (34.1)	
Intra CD8+TIL				
Low	61 (64.2)	53 (86.9)	8 (13.1)	0.004
High	34 (35.8)	20 (58.8)	14 (41.2)	
Peri CD8+TIL				
Low	52 (54.7)	46 (88.5)	6 (11.5)	0.006
High	43 (45.3)	27 (62.8)	16 (37.2)	

WD: Well differentiated; MD: Moderately differentiated; PD: Poorly differentiated.

**Table 3:** Significance of clinical factors and molecular markers by Cox regression analysis.

Variables	Univariate	*p*	Multivariate	*p*
	Odds Ratio (95% CI)		Odds Ratio (95% CI)	
Age	0.991 (0.953–1.031)	0.644	0.994 (0.924–1.069)	0.873
Gender				
Male	1		1	
Female	1.770 (0.622–5.037)	0.285	2.800 (0.415–18.887)	0.290
Lesion site				
Head and neck	1		1	
Acral	1.287 (0.356–4.652)	0.701	3.349 (0.382–29.374)	0.275
Extremity	0.000 (0.000–0.000)	0.995	0.000 (0.000–0.000)	0.998
Trunk	0.000 (0.000–0.000)	0.986	0.000 (0.000–0.000)	0.983
Tumor size, cm				
≤2	1		1	
>2	1.614 (0.598–4.357)	0.345	4.812 (1.025–22.588)	0.046
Histologic grade				
WD	1		1	
MD	1.102 (0.322–3.772)	0.877	3.452 (0.543–21.936)	0.189
PD	3.130 (0.626–15.646)	0.165	13.576 (1.151–160.104)	0.038
COPB2				
Low	1		1	
High	6.734 (2.328–19.478)	<0.001	10.905 (1.714–69.372)	0.011
MHC I				
Low	1		1	
High	3.403 (1.270–9.121)	0.015	3.172 (0.593–16.975)	0.177
MHC II				
Low	1		1	
High	3.701 (1.335–10.261)	0.012	1.849 (0.289–11.830)	0.516
Intra CD4+TIL				
Low	1		1	
High	3.611 (1.305–9.993)	0.013	2.473 (0.375––16.325)	0.347
Peri CD4+TIL				
Low	1		1	
High	0.984 (0.363–2.666)	0.984	0.218 (0.042–1.123)	0.069
Intra CD8+TIL				
Low	1		1	
High	2.798 (1.007–7.773)	0.048	4.186 (0.851–20.579)	0.078
Peri CD8+TIL				
Low	1		1	
High	2.017 (0.692–5.875)	0.199	0.489 (0.083–2.875)	0.429

WD: Well differentiated; MD: Moderately differentiated; PD: Poorly differentiated.

## Data Availability

The data presented in this study are available on request from the corresponding author.
